# Characterization of binding interactions of SARS-CoV-2 spike protein and DNA-peptide nanostructures

**DOI:** 10.1038/s41598-022-16914-9

**Published:** 2022-07-27

**Authors:** Marlen Kruse, Basma Altattan, Eva-Maria Laux, Nico Grasse, Lars Heinig, Christin Möser, David M. Smith, Ralph Hölzel

**Affiliations:** 1 Fraunhofer IZI-BB, Am Mühlenberg 13, 14476 Potsdam, Germany; 2 University of Potsdam, Am Neuen Palais 10, 14469 Potsdam, Germany; 3 Fraunhofer IZI, Perlickstraße 1, 04103 Leipzig, Germany; 4 Preclinics GmbH, Wetzlarer Straße 20, 14482 Potsdam, Germany; 5 Peter Debye Institute for Soft Matter Physics, University of Leipzig, Linnéstr. 5, 04103 Leipzig, Germany; 6 Medical Faculty, Institute of Clinical Immunology, University of Leipzig, Liebigstr. 18, 04103 Leipzig, Germany; 7 Dhirubhai Ambani Institute of Information and Communication Technology, Gandhinagar, 382 007 India; 8 Free University of Berlin, 14195 Berlin, Germany

**Keywords:** Nanostructures, Nanoscale biophysics, DNA nanostructures, Viral proteins, Biological physics

## Abstract

Binding interactions of the spike proteins of the severe acute respiratory syndrome corona virus 2 (SARS-CoV-2) to a peptide fragment derived from the human angiotensin converting enzyme 2 (hACE2) receptor are investigated. The peptide is employed as capture moiety in enzyme linked immunosorbent assays (ELISA) and quantitative binding interaction measurements that are based on fluorescence proximity sensing (switchSENSE). In both techniques, the peptide is presented on an oligovalent DNA nanostructure, in order to assess the impact of mono- versus trivalent binding modes. As the analyte, the spike protein and several of its subunits are tested as well as inactivated SARS-CoV-2 and pseudo viruses. While binding of the peptide to the full-length spike protein can be observed, the subunits RBD and S1 do not exhibit binding in the employed concentrations. Variations of the amino acid sequence of the recombinant full-length spike proteins furthermore influence binding behavior. The peptide was coupled to DNA nanostructures that form a geometric complement to the trimeric structure of the spike protein binding sites. An increase in binding strength for trimeric peptide presentation compared to single peptide presentation could be generally observed in ELISA and was quantified in switchSENSE measurements. Binding to inactivated wild type viruses could be shown as well as qualitatively different binding behavior of the Alpha and Beta variants compared to the wild type virus strain in pseudo virus models.

## Introduction

The pandemic of the severe acute respiratory syndrome corona virus 2 (SARS-CoV-2) has hit the world severely, causing over 6 million deaths until May 2022^1^. While the response was originally focused on prevention by individual precautions (e.g.^2,3^), the development of effective vaccines was a major step towards overcoming the coronavirus disease 19 (COVID-19)^4^. Still, there is evidence that the virus will become endemic^5^, therefore, any deeper understanding of the fundamental behavior of the virus is essential and SARS-CoV-2 will remain a relevant topic for science. While vaccination prevents severe cases of COVID-19^6–8^, the search for effective treatment and therapeutics continues and remains challenging (e.g^9^). The understanding of binding interactions of the spike protein on the surface of the virions to the receptor on the surface cells in the respiratory tract is of essential importance. Here we characterize a peptide mimicking the targeted receptor and its binding to the spike protein under varying circumstances. Different subfragments of the spike protein as well as full-length proteins with and without amino acid modifications are tested for their binding behavior towards peptide-DNA nanostructures, which are used as structural scaffolds for investigating the impact of oligovalent binding modes. Furthermore, we investigate binding of inactivated wild type SARS-CoV-2 and pseudo viruses expressing the wild type and two mutations of the spike protein.

Peptide-based structures that bind virus surface proteins are valuable tools for investigating binding interactions and can potentially be used in therapeutics and diagnostics for viral infections such as COVID-19 as well^10,11^. In particular, peptide fragments that mimic the binding site of the viral protein on the targeted cellular structure can be used as “decoy” moieties to inhibit the attachment and fusion of virus particles with host cells^12–16^, or potentially serve as tools in analytical investigations of specific virus-host interactions^17^. In the simplest form, these comprise the amino acid sequence in the region of the targeted protein, which is directly involved in the binding interaction with the viral protein. For SARS-CoV, such peptides have been derived from the sequence of the human angiotensin converting enzyme 2 (hACE2) receptor. Several hACE2-derived sequences between 23 and 35 amino acids in length, derived by alanine scan mutagenesis analysis, were reported by Han et al.^18^ in 2006, which showed inhibitory activity against SARS-CoV, responsible for the 2002–2003 outbreak, with IC_50_ values ranging from 0.1 to 50 µM. For the currently relevant SARS-CoV-2, *in-silico* methods have been used to assist in the development of peptides^19–22^. The peptide SBP1 was developed by Zhang et al.^21^ from the alpha1 helix sequence of the N-terminal region of hACE2 and is made up of 23 amino acids. The hACE2 constitutes a known receptor for the spike protein of the new SARS-CoV-2^23,24^. According to Zhang et al., SBP1 showed µM binding affinity to the receptor-binding domain (RBD) of SARS-CoV-2 from one supplier^21^. We characterize the peptide further and compare its binding to different subunits of the spike protein, as well as the full-length protein with and without modifications to the amino acid sequence.

Here, we utilize DNA nanostructures as structural scaffolds for enabling the presentation of multiple SBP1 peptides, arranged in an approximate geometric complement to the targeted binding sites on the spike protein. This strategy takes advantage of cooperative, oligovalent binding, which is a fundamental principle in both natural and engineered biological systems to enhance binding affinity between ligands and multimeric receptors^25^. Especially in the case of ligands that bind to virus surface proteins, small DNA-based nanostructures have been used to arrange carbohydrate^26–29^ and peptide^14,15^ moieties, enhancing the overall binding interaction beyond that provided by typically weak monovalent interactions. While in this work, the DNA structure is used as an analytical device to quantify oligovalent binding interactions, a hypothetic therapeutic application of oligovalent DNA nanostructures would require stabilization in order to avoid enzymatic digestion (e.g. Phosphorothioate-Oligonucleotides^30^).

Here a four-arm DNA nanostructure, formed by the hybridization of four, partially complementary DNA oligonucleotides, was employed as a structural scaffold. The DNA nanostructures are a modification of the ones presented by Moeser et al.^31^. In that case, a trimeric DNA structure was used to analyze oligovalent binding of a peptide to EphrinA2 receptors. The authors characterized the DNA structures showing assembly and stability, as well as atomic force microscopy images. Similar characteristics are expected for the adapted structure carrying the SBP1 peptide. The four-arm DNA structure has been previously employed by Kruse et al.^17^ in the case of an influenza A virus-binding peptide. The melting temperature of the fully double stranded structure was calculated to lie at ~ 84 °C.

In our binding assays, the DNA-peptide nanostructures are a substitute for the natural target, here the hACE2 receptor. One arm enables immobilization of the entire construct to a substrate (e.g. gold electrode or surface of an ELISA plate), while the three remaining arms carry the SBP1 peptide. This trimeric peptide presentation is meant to form an approximate geometric complement to the homo trimeric structure of the spike protein of SARS-CoV-2. The binding behavior of the trimer was determined and compared to the binding of single SBP1 peptide to the spike protein.

For the binding measurements we applied two measurement techniques, namely enzyme-linked immunosorbent assays (ELISA) and switchSENSE technology. By employing these two assays, we demonstrate how the concept of DNA-templated oligovalent interactions with virus proteins can be effectively integrated into both, a universally common measurement method available to nearly any biological facility (ELISA) as well as a more specialized, quantitative approach (switchSENSE). Generally, the methods here can be transferred to any analytical method that involves detecting a target analyte in solution using a surface-bound capture moiety, such as surface plasmon resonance (SPR) or even lateral flow assays.

In ELISA, the receptor, in this case the DNA-peptide nanostructure, is immobilized on a surface to act as capture molecule for binding the antigen, which is free in solution. An antibody is subsequently added that also binds the antigen at a different epitope. Then typically a secondary antibody that carries an enzyme is added and binds to the first antibody, if it is available. An enzymatic substance is then added to induce a visible color change. A microplate reader then measures the absorption. The results are interpreted semi-quantitatively. The absorbance signal should increase with increasing concentrations of the added analyte, in the case of a binding event. A higher absorbance signal is interpreted as an enhanced binding. ELISAs can in principle be used to determine quantitative values, but this requires a calibration curve^32^. This was not done here. ELISAs provide reliable, reproducible, economical and controllable results of binding, and are an ubiquitously employed analytical method, here primarily used for a semi-quantitative assessment of binding^33^.

switchSENSE technology on the other hand is a more quantitative technique, that measures association and dissociation rate constants^34,35^. Here the receptor or capture molecule is coupled to a single stranded DNA strand and then immobilized on gold electrodes by conjugate hybridization to an anchored complementary DNA strand. The anchor strand carries a fluorescent molecule at the distal end. A binding event is then detected in real time by a change in the fluorescence intensity upon binding^36^.

## Results and discussion

### Binding of DNA nanostructures to fragments of the SARS-CoV-2 spike protein and to full-length spike proteins with and without amino acid sequence variations

In order to functionalize the DNA strands with SBP1, copper-free click chemistry was utilized. It has proven to provide reliable receptor-mimicking constructs for peptide-virus interactions in previous studies^17^ and is in general favorable for the synthesis of oligovalent DNA-peptide nanostructures^31,37^. The DNA-SBP1 strands were assembled to DNA-SBP1 nanostructures by hybridization of four partially complementary DNA strands using a temperature ramp. Coupling of SBP1 to the DNA strands as well as correct folding of the structures was analyzed by native PAGE (see Supporting Information SI3 and SI4). The DNA nanostructure was optimized for the application in switchSENSE technology as it is made up of three peptide-coupled DNA strands and a fourth strand, that contains the complementary strand to the 48 base long anchor (NL-B48) on the gold electrodes of the measurement chip. The construct can therefore be immobilized by conjugate hybridization on the chip surface. After the hybridization, the protein was injected into the flow channel and binding was detected in the instrument’s static mode (fluorescence proximity sensing). In this measurement mode, the DNA strand is kept in an upright position by an applied negative direct voltage (U =  − 0.1 V). The binding interaction is then observed by a change in the fluorescence intensity that is caused by the close chemical proximity of the binding partner^36^. In this case, the binding resulted in a strong decrease of the fluorescence intensity.

**Figure 1:**
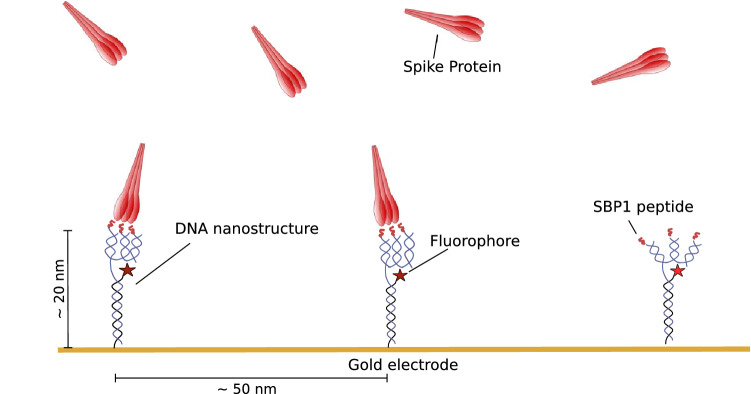
Schematic overview of the experimental setup. Oligovalent DNA structures are immobilized on a gold sensor surface and the binding of spike protein to the oligovalently presented SBP1 peptide is observed. A binding event leads to a reduction of detected fluorescent light intensity. The picture is not drawn to scale.

In a first step, we immobilized the four-arm DNA nanostructure that presents three SBP1 peptides and then observed the binding to the SARS-CoV-2 spike protein. The setup is presented schematically in Fig. 1.

Two different subunits of the SARS-CoV-2 spike protein as well as the full-length protein itself were investigated. The protein has been studied intensively^38^ and relevant regions for binding have been determined. We focused our work primarily on the receptor-binding domain (RBD), subunit 1 (S1) and the full-length protein. Full-length protein in this case refers to the ectodomain (ECD). The spike glycoprotein is divided into the subunit 1 (S1) and the subunit 2 (S2). S1 contains the RBD that has been reported to bind to the hACE2-receptor^39^.

**Figure 2:**
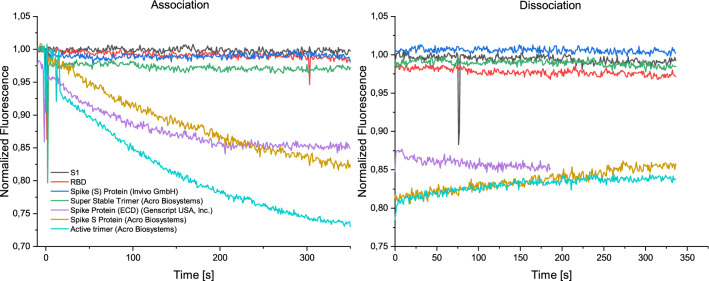
Binding measurements of spike protein and different subunits to SBP1 peptide. Association and dissociation of different subunits of the spike protein, as well as of full-length spike proteins with varying amino acid sequences were analyzed. All proteins were injected at a concentration of 100 nM. The SBP1-peptide-DNA-nanostructure bound the full-length spike proteins from Genscript USA, Inc. and the proteins “SARS-CoV-2 S protein” and “SARS-CoV-2 S protein, active trimer” by Acro Biosystems. The “Super Stable Trimer” by Acro Biosystms and the “Spike (S) Protein” by Invivo BioTech Services GmbH did not show any binding to the peptide. The two proteins have variations in their amino-acid sequences that seem to disturb the binding.

According to Zhang et al., the binding of the SBP1 peptide to the receptor binding domain of the spike protein is weak and for some samples nonexistent. Our experiments confirmed this observation. The peptide presented on the trimeric DNA nanostructure showed no binding to RBD from two different sources up to high concentrations, neither in switchSENSE, nor in ELISA. A similar observation was made for the S1 domain of the spike protein. However, the peptide did bind some of the full-length spike proteins as can be seen in Fig. 2.

Only the full-length protein “SARS-CoV-2 Spike protein (ECD)” of Genscript USA, Inc. and the proteins “SARS-CoV-2 S protein” and “SARS-CoV-2 S protein, active trimer” by Acro Biosystems lead to a strong fluorescence intensity change upon injection.

According to the manufacturers, the proteins were produced in varying expression systems that do not correlate with binding or non-binding behavior of the particular fragments (see “Materials and methods” section, Table 1). The expression system therefore is most likely not responsible for whether a protein binds to the receptor.

**Table 1:** Spike protein fragments and full-length spike proteins and their specifics.

Name	AAS/MW	Modifications	Expression system	Producer
RBD	AA 319–519, ~ 30 kDa	His Tag, Avi Tag	Human	Genscript USA, Inc
RBD	AA 329–538, ~ 30 kDa	His Tag	Drosophila S2	Fraunhofer IZI, Leipzig, Germany
S1	AA 16–685, ~ 79 kDa	His Tag	Human	Genscript USA, Inc
S1	AA 15–682, ~ 100 kDa	His Tag	Drosophila S2	Fraunhofer IZI, Leipzig, Germany
SARS-CoV-2 spike protein (ECD)	AA 16–1213, ~ 135 kDa	His Tag, Flag Tag	Sf9 insect	Genscript USA, Inc
SARS-CoV-2 S protein	AA 16 – 1213, ~ 135 kDa	His Tag	HEK293	Acro Biosystems
SARS-CoV-2 spike (S) protein	AA 14–1213, ~ 137 kDa	His Tag, Mutated polybasic/furin cleavage site to alanine & K986P/V987P	HEK293	Invivo BioTech Services GmbH
Active trimer	AA 16–1213, ~ 138 kDa	His Tag, Trimer motif (26 AA linker) at C-terminus	HEK293	Acro Biosystems
Super stable trimer	AA 16–1213, ~ 138 kDa	F817P, A892P, A899P, A942P, K986P, V987P & alanine substitutions R683A/R685A (furin cleavage site)	HEK293	Acro Biosystems

Curiously, two variations of full-length proteins did not bind to the SBP1-DNA nanostructure: the “SARS-CoV-2 S protein, Super Stable Trimer” by Acro Biosystems and the “Spike (S) protein” from Invivo BioTech Services GmbH. These two proteins have several variations in their amino acid sequences, with both containing identical alanine substitutions at the furin cleavage site (R683A and R685A) and two substitutions to proline at K986P and V987P.

The furin cleavage site is known to influence binding behavior^40,41^. Peacock et al. showed that the polybasic cleavage site is advantageous for human transmission. The variations done on the cleavage site in the two proteins might therefore be a contributing factor for the loss of binding to the hACE2-based peptide.

The amino acids 986 and 987 are located in the region of the Heptad-Repeat Domain 1 (HR-1)^42^. A substitution of these two consecutive amino acids to two prolines hinders a transformation from the prefusion state to the postfusion state^42^. The protein that carries these replacements is named S-2P^43^. The substitution has a stabilizing effect as it conserves the prefusion state of the protein. The stabilization can even be improved as has been done for the “Super Stable Trimer” by Acro Biosystems. Here, four additional amino acids are replaced by proline, namely F817P, A892P, A899P and A942P. This corresponds to the “HexaPro” version of the spike protein described by Hsieh et al.^43^. The reduced conformational flexibility of the stabilized spike protein might negatively influence the binding behavior.

The measurement results show that the variations at the furin cleavage site and the proline substitutions for stabilization of the prefusion state influence the binding of the proteins to the hACE2-derived SBP1-peptide. Since the proteins are modified in both of these aspects, it is not possible to determine, which of these variations causes the ceasing of the binding or if the combination of both variations is responsible. It would be interesting to investigate a protein that carries only one of the variations, either furin cleavage site variation or stabilization of the prefusion state.

The peptide SBP1 mimics the receptor and even though its alpha-helical conformation is supposed to be very similar to that of the original hACE2 binding domain, there can be small conformational differences influencing the binding behavior of the stabilized versions of the spike protein. A further hypothesis would be that the region of the hACE2 receptor, from which the SBP1 peptide was deduced, might play a role in the fusion process.

The results show that relatively small variations of the protein amino acid sequence can drastically affect binding behavior up to the point where no binding occurs.

### DNA nanostructure carrying one or three peptides in comparison

**Figure 3:**
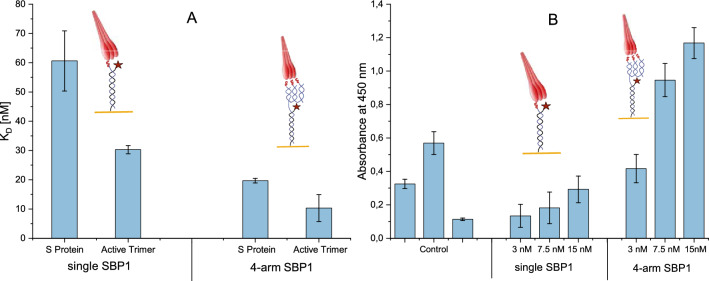
Spike protein binding to three or one peptide per DNA nanostructure. (**A**) Results from the switchSENSE measurements of the dissociation constants K_D_ for the binding interaction of “SARS-CoV-2 S protein” and “SARS-CoV-2 S protein, active trimer” by Acro Biosystems to the monovalent peptide presenting DNA (single SBP1) and the trimeric peptide presenting DNA structure (4-arm SBP1). Trimeric peptide presentation reduces the dissociation constant by a third compared to the monovalent peptide presentation. The “SARS-CoV-2 S protein, active trimer” binds more strongly than the “SARS-CoV-2 S protein”, presumably due to variations in glycosylation and amino acid sequence variations. (**B**) Results from ELISA for a control, immobilized DNA with one peptide or three peptides and subsequent binding analysis of the “SARS-CoV-2 S protein, active trimer”. Here as well the trimeric presentation results in a significant increase of the signal compared to the monovalent peptide presentation. Although the experiment was repeated multiple times, the results shown here come from one well plate to allow for comparability.

To determine the influence of the trimeric structure of the DNA-peptide-nanostructure, we compared the binding of the full-length spike proteins “SARS-CoV-2 S protein” and “SARS-CoV-2 S protein, active trimer” both by Acro Biosystems to the DNA nanostructure carrying three peptides or just a single peptide using the switchSENSE technology and ELISA. For the switchSENSE experiments, similar experimental parameters were applied to the interaction measurement between a mono- and trivalent SBP1 presentation on the DNA nanostructure as were chosen in “Binding of DNA nanostructures to fragments of the SARS-CoV-2 spike protein and to full-length spike proteins with and without amino acid sequence variations” section. Dissociation times were increased to t = 1200 s and the concentration of spike proteins was varied between 25 and 200 nM. After each binding experiment, the surface was regenerated with fresh DNA-peptide nanostructures since complete dissociation was not reached. As negative control, only the DNA nanostructure without any peptide conjugated was immobilized. The measurement parameters were identical for all measurements. The resulting graphs (see Supporting Information SI1) were then fit with a global mono-exponential function according to standard binding affinity theory^44^ in order to determine kinetic rates and dissociation constant K_D_ as can be found in Fig. 3A.

The experiments were confirmed by ELISA for DNA nanostructures holding three, one and zero peptides, and varying concentrations of “SARS-CoV-2 S protein, active trimer” from Acro Biosystems (see Fig. 3B).

Figure 3A shows the results of the determination of dissociation constants K_D_ for the binding of the “SARS-CoV-2 S protein” and “SARS-CoV-2 S protein, active trimer” by Acro Biosystems to the monovalent peptide presentation (single SBP1) and the trivalent peptide presentation (4-arm SBP1) measured in switchSENSE and ELISA.

As can be seen in Table 2, the dissociation constant of one SBP1-peptide per DNA nanostructure is around three times higher than that of the trivalent peptide-DNA nanostructure for both proteins. It was expected that the oligovalent peptide presentation on the four arm nanostructure would increase affinity (e.g.^31,45^).

**Table 2:** Dissociation constants for monovalent and trivalent peptide presenting DNA nanostructures to two spike proteins.

	K_D_ (nM) monovalent peptide presentation (single SBP1)	K_D_ (nM) trivalent peptide presentation (4-arm SBP1)
SARS-CoV-2 S protein	60.1 ± 10.2	19.7 ± 0.8
SARS-CoV-2 S protein, active trimer	30.3 ± 1.4	10.3 ± 4.6

In the ELISA, the binding of “SARS-CoV-2 S protein, active trimer” by Acro Biosystems to the one and three peptide-carrying DNA was analyzed. For constructs carrying three peptides, the absorbance showed almost three times the signal strength as compared to the constructs holding one peptide. Nanostructures carrying zero peptides were used as control to confirm specific binding to the hACE2 derived peptide, and exclude unspecific binding to the DNA nanostructures.

The experiments were repeated several times. The spike proteins “SARS-CoV-2 S protein” and “SARS CoV-2 S protein, Active Trimer” were handled according to the manufacturer’s instructions. The lyophilized protein was first resuspended in ddH2O, then aliquoted and finally stored at − 80 °C until use. Nevertheless, it was observed that over time, the activity of the proteins seemed to degrade, which has also been reported in literature^43^. This degradation resulted in variations of the measured binding affinity and therefore effected the error. Still, we were able to differentiate between the one and three peptides per nanostructure. The trimeric presentation of the peptide always resulted in lower dissociation constants than the monomeric presentation.

### DNA-SBP1 nanostructures binding to inactivated viruses

**Figure 4:**
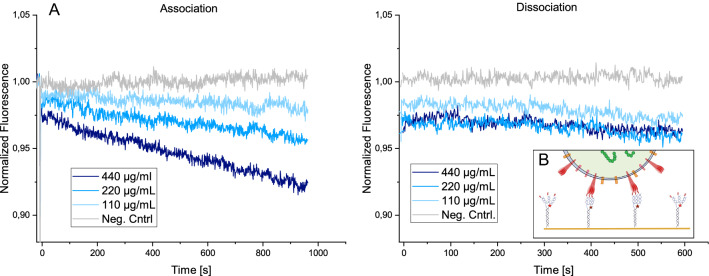
SARS-CoV-2 virus material binding to SBP1-DNA nanostructure. (**A**) Association and dissociation of inactivated SARS-CoV-2 to the DNA nanostructure carrying three SBP1 peptides are presented. The association shows a concentration dependent decrease of the fluorescence intensity signal, indicating a correct binding interaction. The dissociation shows almost no signal change presumably due to multivalent binding interactions. (**B**) Schematic overview of binding process, not drawn to scale.

In order to prove that the DNA-peptide-nanostructures bind not only to the individual recombinant spike protein floating freely in solution, but also to the protein as it is found on the surface of the virus, the binding to whole SARS-CoV-2 particles was measured. Viruses were chemically inactivated by ß-propiolactone in order to adhere to biosafety requirements. The DNA construct that displayed three peptides was employed as it had shown to be the strongest binder. The DNA-SBP1 nanostructure was again immobilized by complementary hybridization. Subsequently inactivated SARS-CoV-2 sample was injected at varying concentrations. The virus particles have an average diameter of around 90 nm^46^, as was also verified by dynamic light scattering (see Supporting Information SI2). The average distance between the DNA nanostructures on the surface of the gold electrode is 50 nm^36^, therefore, one virus particle can bind to more than one DNA-peptide nanostructure (see Fig. 4B). The result of the binding experiment can be seen in Fig. 4A.

Figure 4A shows the association and dissociation of the inactivated virus from the DNA-peptide nanostructures. During association, a concentration dependent decrease of fluorescence intensity was observed. The signal change is more linear than exponential. This has been described before for multivalent virus-receptor interactions^47^. The dissociation shows almost no signal change over the measurement time of around 600 s. This indicates a strong binding interaction, which can be attributed to the high affinity of the individual DNA-peptide nanostructures to the spike protein as well as multivalent binding effects to multiple DNA-peptide nanostructures on the surface, which has been reported for previous virus-receptor interaction studies^17^. As a negative control, the ssDNA (cNLB48) that is complementary to the anchor strand (NL-B48) and that carries no peptide was hybridized onto the sensor surface and virus solution at a concentration of 220 µg/mL was injected. No signal change was observable upon injection.

#### DNA-SBP1 nanostructures binding to pseudo viruses

**Figure 5:**
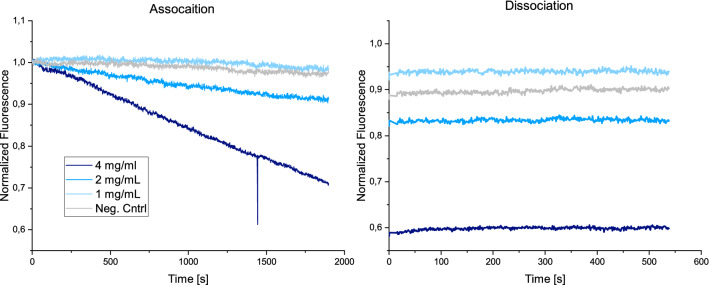
Pseudo virus—peptide interaction. Lenti-virus based pseudo viruses that carry the SARS-CoV-2 spike protein on their surface were injected and their binding to the DNA nanostructures with three SBP1 peptides was observed. Here as well, concentration dependent linear signal changes in the fluorescence intensity upon injection can be observed in the course of the association.

Further experiments were performed on pseudo virus models for SARS-CoV-2. These use Lenti-viruses as a basis, which are genetically modified to express the spike protein on their viral membrane. The pseudo viruses were also ß-propiolactone inactivated and purified prior to the measurement. Then the three SBP1-peptides carrying DNA structures were once again immobilized on the gold electrodes and the pseudo virus solution was injected. The results of the measurements can be found in Fig. 5.

Figure 5 shows the association and dissociation of the pseudo viruses to and from the three-armed DNA-SBP1 nanostructure. Here as well, a strong linear fluorescence intensity change upon injection of the pseudo virus samples could be seen. The concentrations utilized here were higher than in the case of the inactivated original viruses in “DNA-SBP1 nanostructures binding to inactivated viruses” section. This might be due to differences in sample preparation and varying characteristics of the samples as for example the density of spike proteins on the viral surface or varying surface proteins for pseudo viruses and original viruses. After their production, the pseudo viruses were purified, but it is expected that proteins and other residues from the cell medium remained in the sample solution. For the original viruses, which were purified by ultracentrifugation and were delivered in buffer, the purity of the sample was expected to be higher.

The dissociation showed almost no signal change for any of the injected samples. This indicates again a strong binding over the measurement time. Similar to the original viruses, multivalent binding to multiple DNA nanostructures on the surface of the electrode was expected. As a negative control, DNA without peptide (cNLB48) was immobilized and 2 mg mL^−1^ pseudo virus solution was injected into the channel. There was almost no signal change for the association or the dissociation indicating that the pseudo viruses specifically bound the peptides on the nanostructure. Concentrations below 1 mg mL^−1^ virus solution resulted in signal changes that were no longer distinguishable from the control.

#### DNA-SBP1 nanostructure binding to pseudo virus variants

**Figure 6:**
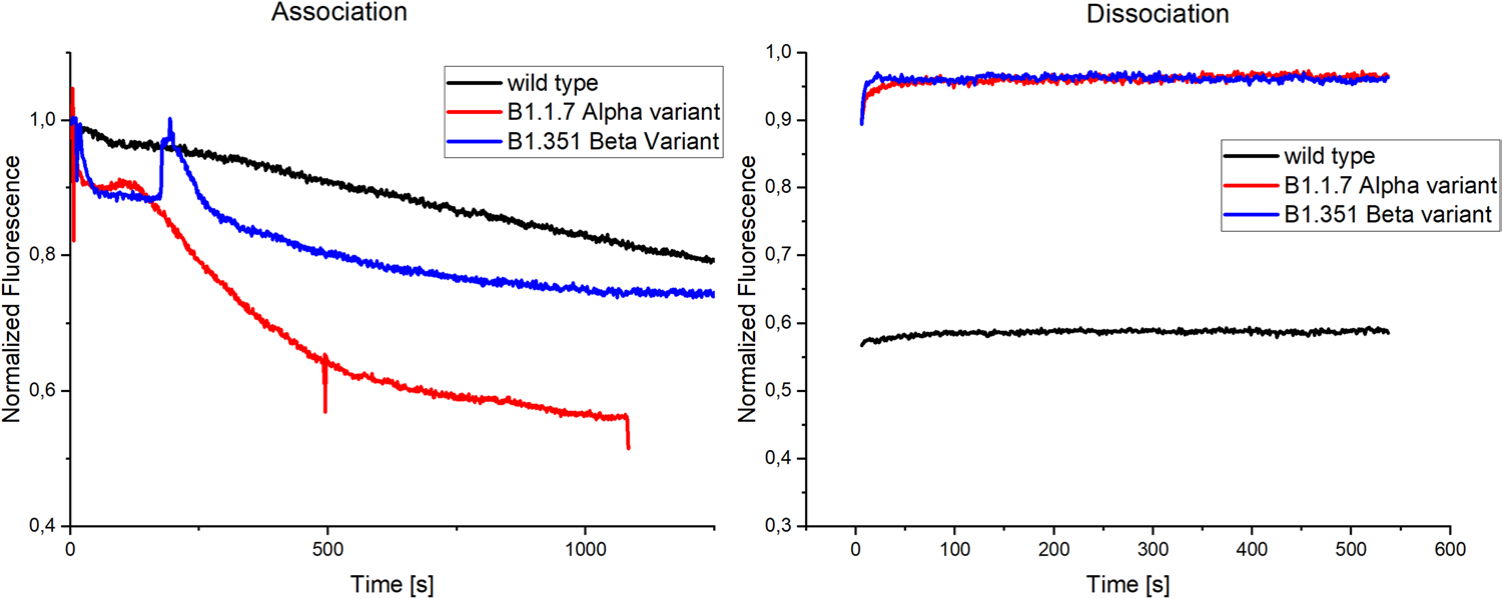
SARS-CoV-2 pseudo virus variants binding to SBP1-peptide. The binding of pseudo viruses that carry spike proteins of three different variants of the SARS-CoV-2 virus to the DNA-peptide nanostructure was determined. Association behavior differed for all three variants. While the wild type showed the typical linear association behavior, Alpha and Beta presented an exponential binding behavior. Spikes in the signal can be attributed to air in the channel system. The Alpha variant induced the strongest signal change over the measurement time. The dissociation displayed almost no signal change for all three samples.

The spike protein on the pseudo viruses can be modified to existing variants of the SARS-CoV-2. The different binding behaviors of the various strains can therefore be analyzed. Here we tested three different spike protein variants namely the wild type (first detected in Wuhan, China), the B1.1.7 or Alpha variant (first detected in the United Kingdom) and the B1.351 or Beta variant (first detected in South Africa)^48^. The results of the binding interaction measurements indicate a substantially different binding behavior of the variants from the wild type. Although the measurements were not quantitative, a qualitative comparison was possible. The results can be seen in Fig. 6.

In Fig. 6, the association and the dissociation of the three pseudo virus samples and their binding to the DNA-SBP1 nanostructures is presented. The association showed strong signal changes for all three samples. The injection of the wild type leads to the typical linear binding behavior, while the injection of the Alpha and Beta variant resulted in exponential binding signals. The Alpha variant showed the fastest and strongest association of the three samples although the protein concentration (c) was the lowest (c_wild_ ~ 4 mg mL^−1^, c_Alpha_ ~ 3 mg mL^−1^, c_Beta_ ~ 4 mg mL^−1^). The spikes in the graph can be attributed to air in the tubing. For the dissociation, almost no signal change was visible for all three samples. This again indicates a strong binding behavior.

While the measurements were repeated and the findings could be reproduced, the influence of production and sample differences has to be considered. The different binding behavior of the variants could be an indication for advantageous mutations for the SARS-CoV-2 variants. The findings need further investigations and should be expanded to other variants.

## Conclusion

The measurements show that the SBP1 peptide specifically binds distinct full-length spike proteins of SARS-CoV-2. While subunits like the receptor binding domain (RBD) and S1 do not show any binding to the peptide, the unmodified full-length spike proteins bind strongly. A variation of the amino acid sequence at the C-Terminal as was done for one spike protein even increases binding. Substitutions to alanine at the furin cleavage site and variations for stabilization of the prefusion state of the protein on the other hand lead to reduced binding. The measurements suggest that even small variations of the amino acid sequence of the spike protein strongly influence the binding process to the hACE2-derived peptide.

The peptide was coupled to DNA nanostructures that mirror the homo trimeric structure of the spike protein. DNA nanostructures carrying three peptides showed the strongest binding affinity towards the full-length spike proteins “SARS-CoV-2 S protein” and “SARS-CoV-2 S protein, active trimer” by Acro Biosystems. Measurements with one peptide per construct resulted in binding of the protein, but, as expected, the affinity was lower. Influences of the protein degradation have to be considered and lead to measurement errors in the quantitative analysis.

The DNA nanostructure carrying three peptides was furthermore utilized as a ligand for measuring interactions with original SARS-CoV-2 and pseudo viruses. Strong concentration dependent binding interaction signals were visible for the associations. No dissociations were measurable presumably due to multivalent binding and rebinding effects of virus particles to the DNA-peptide nanostructures. Variations of the spike protein on the pseudo virus surface lead to a change in binding behavior. While the wild-type spike protein on Lentivirus based pseudo viruses lead to a linear signal change, spike proteins from the Alpha and Beta variants showed a more exponential behavior. While the findings are purely qualitative, they might point towards a change in binding behavior with possible consequences for the transmissibility and virulence of the variants.

## Materials and methods

### SBP1-peptide

The peptide SBP1 was developed by Zhang et al.^21,22^ from molecular dynamic simulations and cryo electron microscopy of the α1 helix sequence of the human angiotensin-converting enzyme 2 (hACE2). The peptide is 23 amino acids long (IEEQAKTFLDKFNHEAEDLFYQS). The peptide was adjusted to carry an azidobutyric acid and 16 PEG-linkers at the N-terminal and it was purchased from Peptide Speciality Laboratories GmbH (Heidelberg, Germany). SBP1 has a molecular mass of 3202 Da.

### DNA-peptide nanostructure

DNA-peptide conjugate preparation is based on previously described protocols^17,31,37^. All DNA strands as presented in Table 3 were purchased from Biomers.net GmbH. Some strands were purchased with an amino group on the 5′ end. These DNA strands were then modified with a dibenzocyclooctyne group (DBCO)-NHS ester (Jena Bioscience GmbH, Jena, Germany) through NHS ester reaction. This allows for the azide-modified hACE2-derived peptide to be conjugated to the DBCO moiety on the oligonucleotide strands, through strain promoted azide-alkyne cycloaddition (SPAAC).

**Table 3:** Oligonucleotide sequences to form DNA nanostructures.

Name	Sequence 5′ → 3′	Modification
p-cNL-B48	acacacacacaactaatcagcgttcgatgcttccgactaatcagccatatcagcttacgacta	5′ NH_2_
n	atttagtttctatca	none
n*o	tgatagaaactaaatataatatgcgagcca	5′ NH_2_
o*p*	tggctcgcatattattagttgtgtgtgtgt	5′ NH_2_
NL-B48	tagtcgtaagctgatatggctgattagtcggaagcatcgaacgctgat	5′ Biotin

Part n is complementary to part n*; part o to part o *; p to p* and cNL-B48 is complementary to NL-B48, which is already immobilized on the gold electrode of the switchSENSE device (without Biotin). For ELISAs, NL-B48 was ordered with 5´ Biotin to attach it to NeutrAvidin on the ELISA plate.

Concentration and purity of unmodified and modified ssDNA and DNA structures were determined using a NanoDrop UV/Vis spectrophotometer at 260 nm.

NHS ester reaction was carried out to attach a functional DBCO group to the DNA for later addition of the peptide through copper-free click chemistry reaction. DBCO-NHS ester was dissolved in DMSO to a concentration of 10 mM. 10% of total volume of 10 × PBS was added to amino-coupled oligonucleotides to a final concentration of 1 × PBS, followed by the addition of DBCO-NHS ester in 100 × molar excess compared to the DNA. NHS ester-activated compounds react with amino groups to yield stable amide bonds and the reaction releases N-hydroxysuccinimide (NHS). The solution was incubated overnight at room temperature and purified the next day through ethanol precipitation to remove excess DBCO-NHS ester.

For the conjugation of the DBCO modified oligonucleotides with the hACE2 derived peptide, SPAAC was performed. DBCO is a cyclooctyne derivative, which reacts with azide-coupled peptides to form a stable triazole. The peptide was dissolved in MilliQ water to a concentration of 2 mM. Afterwards, the peptide was added to DBCO-modified DNA in 1 × PBS at an excess between 15x and 30x. The conjugate solution was left to incubate overnight at room temperature and purified the next day through ethanol precipitation to remove excess peptide.

The DNA nanostructures were formed using partially complementary single stranded DNA, and folded in a thermocycler in 1 × PBS. First, the samples were incubated at 54 °C for 15 min, then incubated at 30 °C for 5 min, and then cooled to 4 °C.

### Spike proteins

The spike proteins were all used according to the manufacturer’s instructions without further purification.

The molecular weights (MW) of the monovalent proteins as given in Table 1 were used to calculate protein concentrations for binding affinity measurements in “Binding of DNA nanostructures to fragments of the SARS-CoV-2 spike protein and to full-length spike proteins with and without amino acid sequence variations” and “DNA nanostructure carrying one or three peptides in comparison” sections.

### Pseudo viruses

Pseudo viruses from preclinics GmbH (Potsdam, Germany) were used. An overview of the production process is given.

#### Cell culture

293T (ATCC, USA) and 293T-hACE2 (Integral Molecular Inc., Philadelphia, PA, USA) were used for pseudo virus creation and testing. Both strains were grown in DMEM (Corning, USA) supplemented with heat inactivated 10% FCS (Sigma-Aldrich, Darmstadt, Germany) and 1% penicillin/streptomycin (Corning). 293 T-hACE2 cells were additionally supplemented with 10 mM HEPES (Corning) and 0,5% puromycin (LEXSY). Cells were grown at 37 °C in a humidified incubator (Sanyo) with 5% CO_2_. They were removed and split with accutase (Corning) followed by centrifugation at 4000 rpm for 5 min with a Megafuge 1.0R (Heraeus, Hanau, Germany). Cells were counted with an automatic cell counter (EVE, NanoEnTek) and plated out according to the test conditions.

#### Generation of different pseudo-viruses

Various pseudo viruses were developed. Each of the pseudo viruses contains a GFP or firefly luciferase (fluc) reporter gene, a lentivirus backbone, and Sars-CoV-2 spike proteins. We followed the ViraSafe^Tm^ Lentiviral Packing System protocol from Biocat GmbH (Heidelberg, Germany) for pseudo virus generation.

The lentivirus backbone is from the third generation and can be processed under S2 conditions. Vectorbuilder GmbH (Neu-Isenburg, Germany) and SinoBiological Europe GmbH (Eschborn, Germany) delivered various plasmids containing the spike protein information. After transfection into 70–80% confluent 293 T cells, pseudo viruses were released into the supernatant and were harvested 36–48 h later and stored at 4 °C.

#### Pseudo virus concentration

All pseudo virus supernatant from the same spike plasmid were pooled and collected. All supernatants were centrifuged at 4000 rpm for 5 min to remove any remaining cell fragments, followed by sterile filtration with a 1.2 µm and a 0.2 µm filter (both filters: Sartorius GmbH, Göttingen, Germany). The sterile fraction was collected and concentrated with a 100 MWCO Spin-X UF centrifugal concentrators (Corning, USA) at 4000 rpm for 30 min.

#### Virus testing and titer determination

The virus titer was determined as relative activity via firefly luminescence measurement. 293 T-hsACE2 cells were seeded into a 96 well plate (Corning) at a density of 1*10^5^ cells per well. After 5 h, all cells were adherent and were inoculated with the pseudo virus solution. Various dilutions from 1 to 10^−9^ were used to determine the virus concentration. 48 h post infection, the supernatants were removed and the cells were lysed inside the well with 25 µl of cell lysis buffer (PJK Biotech GmbH, Kleinbittersdorf, Germany) and incubated for 1 h at room temperature. All subsequent processing of samples was done using the beetle-juice luciferase assay firefly (PJK Biotech GmbH). All samples were analysed in a Mithras LB940 microplate reader (Berthold Technologies GmbH & Co.KG) at 560 nm.

#### Pseudo virus inactivation

The pseudo viruses were treated with ß-propiolactone (Serva Electrophoresis GmbH, Heidelberg, Germany) for inactivation. To validate the efficiency, a stock solution was divided into an untreated fraction (control) and a fraction, which was treated with 0.1% ß-propiolactone. After 16 h of incubation at 4 °C, samples were transferred to 37 °C for 2 h to hydrolyze all residual ß-propiolactone to prevent cytotoxicity to mammalian cells^49^. After hydrolysis, samples were inoculated onto 293T-hsACE2 cells, and incubated at 37 °C with 5% CO_2_ in a humidified incubator. 48 h after infection, the cells were observed via luminescence detection in order to determine the infection rate.

### Inactivated SARS-CoV-2

Inactivated SARS CoV-2 were provided by Fraunhofer Institute for Cell Therapy and Immunology, Leipzig, Germany. They were ß-propiolactone inactivated and suspended in MES buffer. The concentration prior to inactivation was ~ 1.5 × 10^6^ PFU/mL. The sample was kept at − 80 °C until further use.

### SwitchSENSE assays

For binding affinity measurements, a DRX^2^ instrument (Dynamic Biosensors GmbH, Martinsried, Germany) was employed. The method was used according to standard operating procedure as described by Müller-Landau et al.^36^. The DNA-peptide nanostructures were immobilized via conjugate hybridization at a concentration of 500 nM over a period of 360 s. All binding affinity measurements were conducted in static mode. As running buffer, PE140 (10 mM Na_2_HPO_4_/NaH_2_PO_4,_ 140 mM NaCl, 0.05% Tween20, 50 µM EDTA, 50 µM EGTA) was utilized and PE40 (10 mM Na_2_HPO_4_/NaH_2_PO_4,_ 40 mM NaCl, 0.05% Tween20, 50 µM EDTA, 50 µM EGTA) was utilized as auxiliary buffer. All analytes were diluted in PE140 for measurements.

All measurements were carried out on standard multipurpose biochips MPC-48–1-R1-S (Dynamic Biosensors GmbH) at 25 °C system temperature. Prior to each interaction measurement, a passivation of the channel by injection of thiol-containing passivation solution (Dynamic Biosensors GmbH) was performed to avoid non-specific binding. Only electrodes that showed a fluorescence amplitude of > 50 kcps in the subsequently performed status tests were used for measurements.

For measurements with spike protein or fragments of the spike protein, an analyte concentration between 25 and 500 nM was chosen. After each interaction measurement the surface was regenerated. The association volume of the protein was chosen at 300 µL and the flow rate was set at 50 µL min^−1^ for 6 min. The dissociation volume was chosen at 20 mL and the flow rate at 1 mL min^−1^ for 20 min using the peristaltic pump.

For the measurements with inactivated virus material or pseudo viruses the parameters for the interaction were chosen differently. The association volume was 100 µL and was pumped at a flow rate of 5 µL min^−1^ for 20 min. The dissociation volume contained 10 mL and had a flow rate of 1 mL min^−1^ for a time of 10 min using the peristaltic pump. Here as well, the surface was regenerated after each interaction measurement.

### ELISA assays

A functional ELISA was performed to analyze the binding parameters (K_D_ value, B_max_ value, linear dynamic range). First, a 96-well microtiter plate (Nunc maxisorp, transparent; Thermo Fisher Scientific, USA) was coated with 0.5 µg/well NeutrAvidin (Thermo Fisher Scientific, USA) in 0.1 M NaHCO_3_ buffer (pH 8.0) and incubated at 4 °C overnight. The plate was washed three times with wash buffer (1 × PBS, 1 mM MgCl_2_, 0.1% Tween 20, pH 7.4) and 1 µg/well (100 µL/well) of DNA nanostructures was added and incubated for 60 min at room temperature with gentle shaking. The supernatant was then discarded, and the plate was washed three times with 200 µL of blocking buffer (1 × PBS, 1 mM MgCl_2_, 0.1% Tween 20, 1% BSA, pH 7.4). A 1:2 dilution series of SARS-CoV 2 protein in blocking buffer, between 100 and 0.05 nM was then added and incubated for an additional 60 min at room temperature.

The supernatant was again discarded and the plate was washed three times with blocking buffer. Subsequently, a 6 × anti-His-Tag antibody (Thermo Fisher Scientific, USA) was diluted 1:500 in blocking buffer and 100 µL/well was added. The plate was incubated for 1 h at room temperature in the dark. It was finally washed three times with blocking buffer and 100 µL/well of TMB substrate solution (3,3',5,5'-Tetramethylbenzidine, Pierce™ TMB Substrate Kit—Thermo Fisher Scientific, USA) was added and incubated for 20 min at room temperature in the dark, during which the color of the solution changed from colorless to blue. The reaction was stopped by adding 50 µL/well of 2 NH_2_SO_4_, while the color changed from blue to yellow and the absorbance was measured in a microplate reader (Tecan, Germany) at 450 nm and at 520 nm; the latter value was subtracted as a reference.

## Supplementary Information

Supplementary Information.

## Data Availability

The datasets generated during and analyzed during the current study are available from the corresponding author on reasonable request.
