# Supplementary material for: Characterization of binding interactions of SARS-CoV-2 spike protein and DNA-peptide nanostructures
Source: Sci Rep. 2022 Jul 27;12:12828. doi: 10.1038/s41598-022-16914-9 (PMC9328006; doi:10.1038/s41598-022-16914-9)
Supplement: Supplementary file 1 — Supplementary Information. [file 41598_2022_16914_MOESM1_ESM.pdf]

Supporting Information for: Characterization of binding interactions of SARS-CoV-2 spike protein and DNA-peptide nanostructures

SI1: Measurement and fit curves for Spike Protein Active Trimer binding to monovalent and trivalent DNA-SBP1 nanostructures.

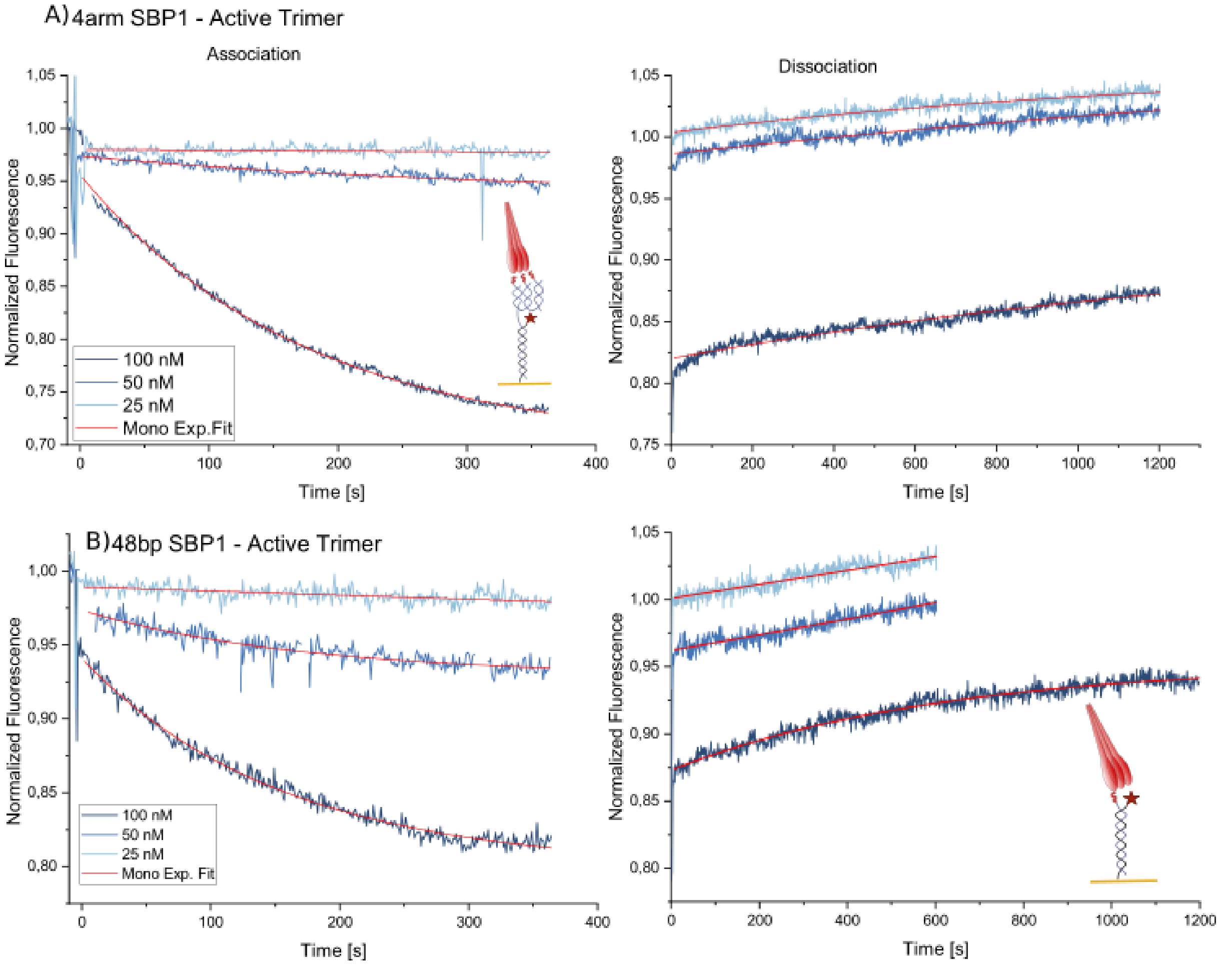

Figure 1 Binding curves of spike protein binding to monovalent and trivalent peptide presentation in DRX[2]

The graphs correspond to the measurements of "SARS CoV-2 S Protein, active trimer" (Acro Biosystems) to the trivalent and the monovalent peptide presenting DNA nanostructures in switchSENSE technology. The red graphs are global mono exponential fit curves. Using standard binding interaction theory [1] k_{on}, k_{off} and K_D were calculated and can be found in Table 1 of the main manuscript.

SI2: Size distribution of inacativated SARS-CoV-2 viruses determined by dynamic light scattering

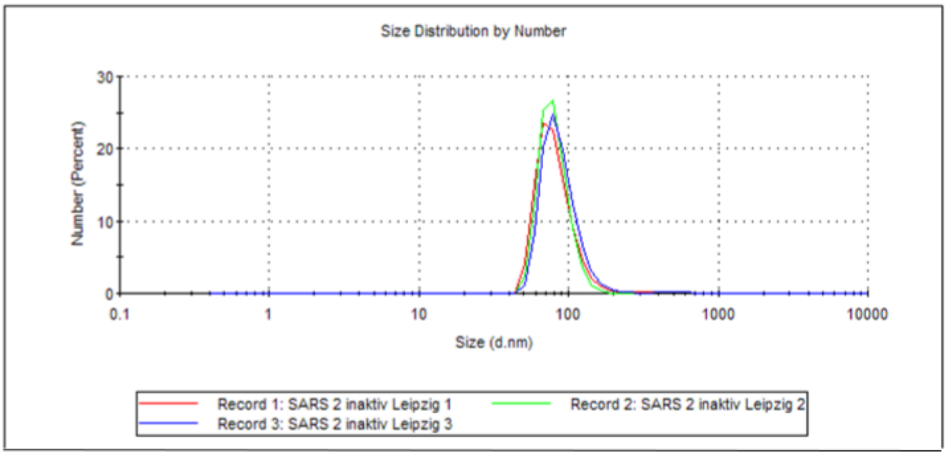

Figure 2 Result of size distribution analysis of the inactivated SARS-CoV-2 sample.

The size distribution indicates a maximum at d= (82 ± 25) nm. The measurement was performed using undiluted sample. As the sample was suspended in buffer, the viscosity of the dispersant was set to 0.8872 cP. The result indicates the expected size of around 90 nm as it is reported in literature[2]. The sample contained larger agglomerates that influenced the result and error of the dynamic light scattering measurement.

SI3: Individual DNA strands analyzed by native PAGE

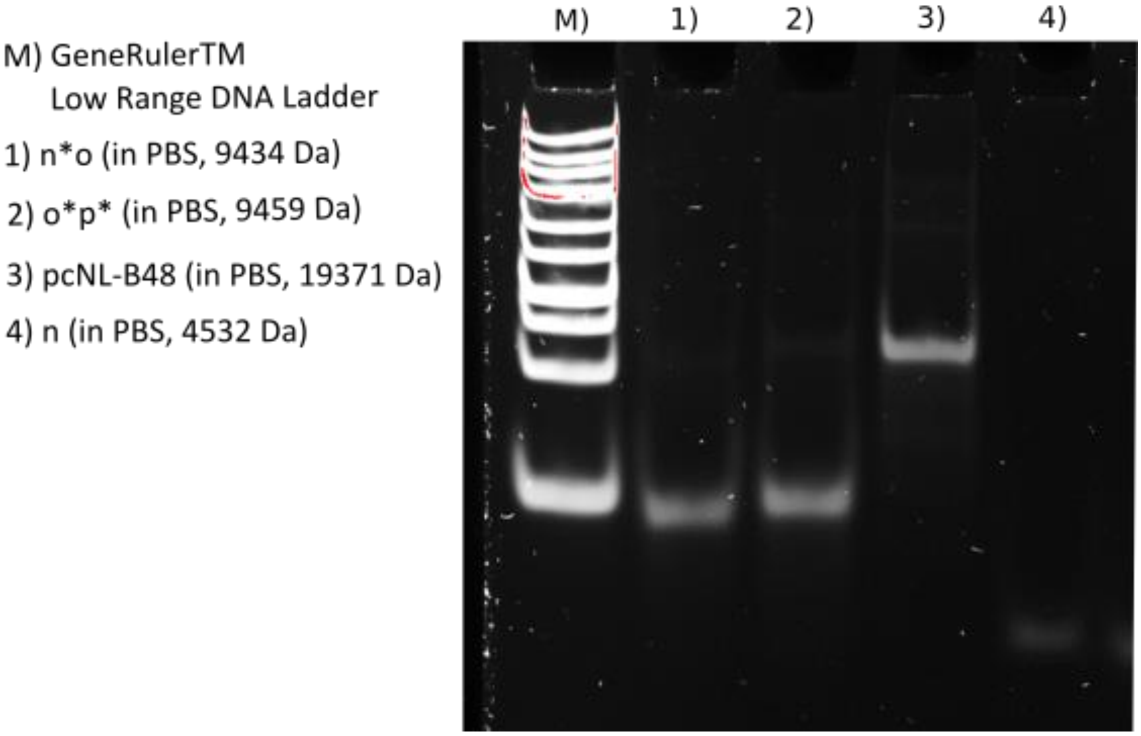

Figure 3: Native PAGE of DNA single strands prior to self-assembly. Gels were imaged using the ChemiDoc MP Imaging System with UV excitation (302 nm) and 590/110 nm emission. Gel image was cropped, edited (brightness + 40 %, contrast + 20 %) and mirrored. The original image can be seen in Figure 4.

In order to characterize the DNA nanostructure, the individual strands were analyzed by native PAGE. The resulting image shows bands that correspond well with the calculated molecular weight. The shortest strand n in line four is only faintly visible due to the lower molecular weight. The DNA strands are partly complementary, which is indicated by an asterisk: n* is complementary to n etc. The 12% (v/v) gel was stained with SYBR® Gold Nucleic Acid Gel Stain [Thermo Fisher Scientific, Waltham, MA, USA] and imaged under UV light. GeneRulerTM Low Range DNA Ladder [Thermo Fisher Scientific, Waltham, MA, USA] served as control, not as ruler.

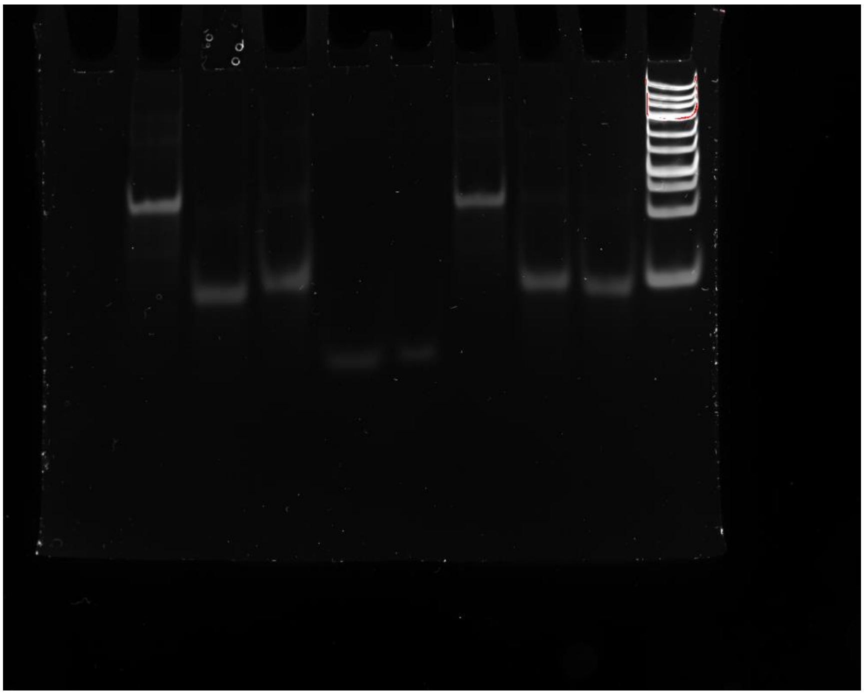

Figure 4: Original image form which Figure 3 was prepared. Additional bands are not relevant to this work.

SI4: Self-assembly of partially complementary DNA strands shown by native PAGE

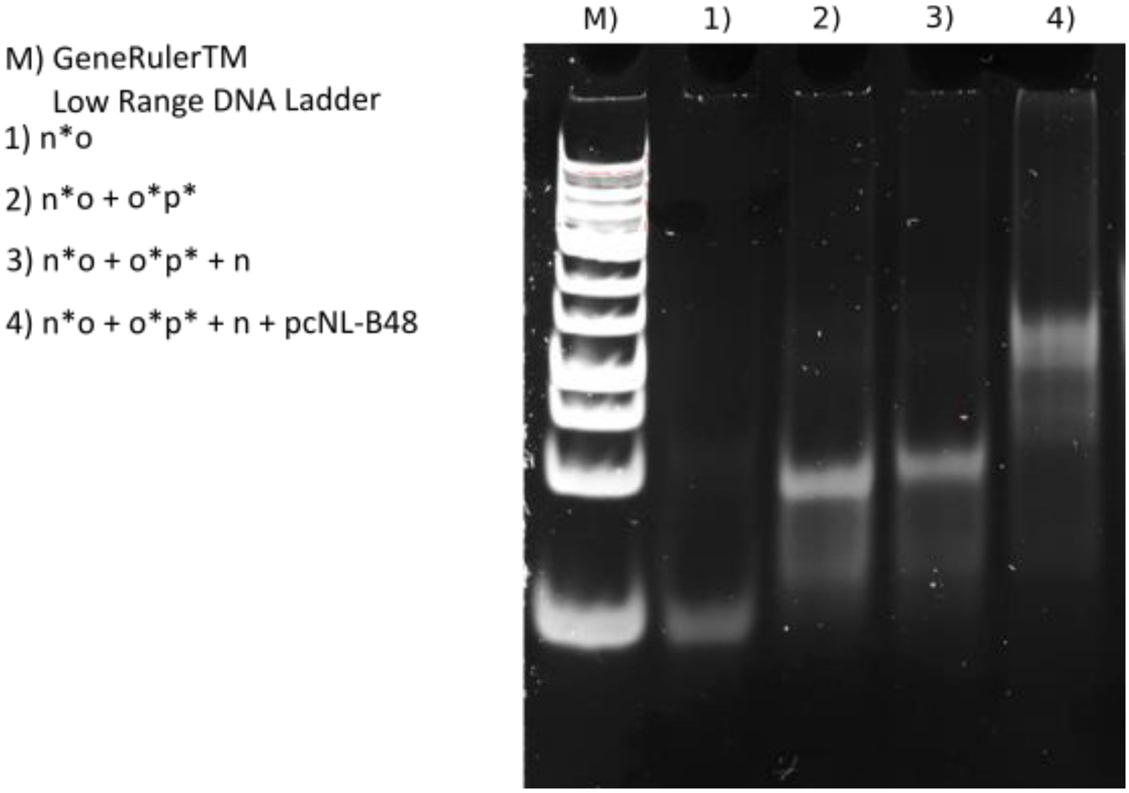

Figure 5: Stepwise formation of the four arm DNA nanostructure as analyzed by native PAGE. Gels were imaged using the ChemiDoc MP Imaging System with UV excitation (302 nm) and 590/110 nm emission. Gel image was cropped and edited (brightness + 40 %, contrast + 20 %). Original image can be found in Figure 6.

The stepwise assembly of the DNA nanostructure was analyzed by native PAGE. As can be seen in Figure 5, folding of the structure appears according to the expected molecular weight increase. The structures were folded in 1x PBS using a thermocycler. The 10% (v/v) gel was stained with SYBR® Gold Nucleic Acid Gel Stain [Thermo Fisher Scientific, Waltham, MA, USA] and imaged under UV light. GeneRulerTM Low Range DNA Ladder [Thermo Fisher Scientific, Waltham, MA, USA] served as control, not as ruler.

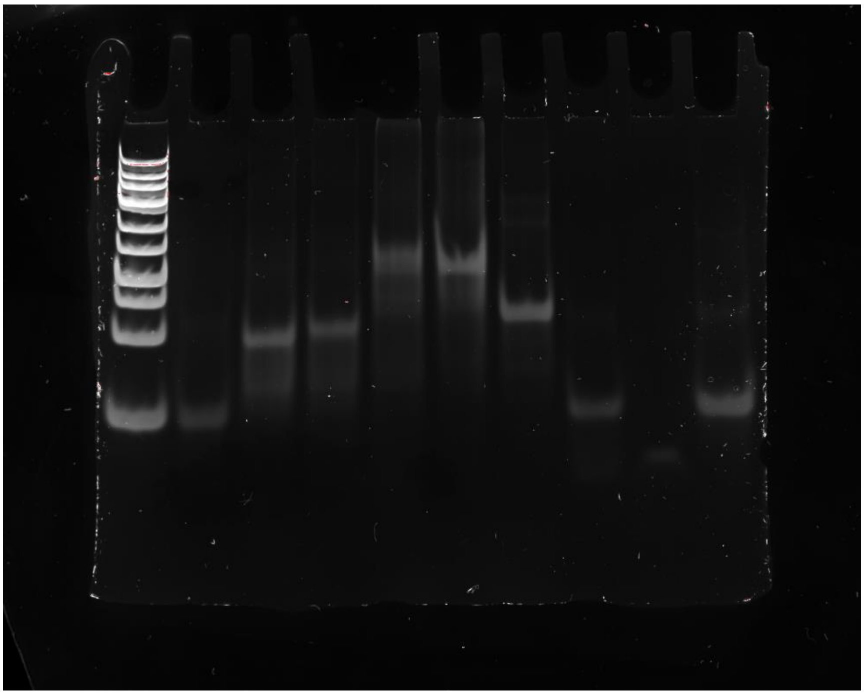

Figure 6: Orginal image from which Figure 5 was prepared. Additional bands are not relevant to this work.

References

1. Goodrich, J. A. & Kugel, J. F. *Binding and Kinetics for Molecular Biologists* (CSH Press, 2007).
2. Klein, S. *et al.* SARS-CoV-2 structure and replication characterized by in situ cryo-electron tomography. *Nature communications* **11,** 5885; 10.1038/s41467-020-19619-7 (2020).
